# Focal Infection: Its Prevalence and Control*Read before the University of Michigan Dental Society, April 21, 1920.

**Published:** 1920-05

**Authors:** M. Rattner


### FOCAL INFECTION: ITS PREVALENCE AND CONTROL *

BY M. RATTNER

### HISTORY

Systemic disease or ill health with its relation to oral infection has been known since dentistry was first practiced. Although nothing was known about infection or bacteria up to the time of Pasteur, yet there are many references to this connection in the literature of the ancients. As far as surgery is concerned, Lister, in 1867, definitely proved the deadliness of infection and started the medical and dental world upon a new basis.

The first definite writing that we have in the United States upon ill health as caused by carious teeth was that of Dr. Benjamin Rush, of the University of Pennsylvania, in 1801. He said, "I have been made happy by discovering that I have only added to the observations of other physicians, in pointing out a connection between the extraction of decayed and diseased teeth and the cure of general disease."

What American dentistry, which was the most progressive from 1840 on, was most interested in was the technical department, consisting largely of prosthesis and the restoration of decayed teeth with gold or amalgam fillings. Little else was deemed necessary. What, then, has caused this untoward excitement that we now hear about as focal infection? It was left for an English physician, Dr. Hunter, to put American dentistry on its mettle by saying that American dentistry, through technical efforts in the restoration of defective teeth with inlays, fillings, bridges and artificial dentures, was the direct

---

* Read before the University of Michigan Dental Society, April 21, 1920.

cause of anemia, septicemia, heart lesions and many other systemic ills.

A new era now arose. Restorations were put in the background to some extent and research instituted. This latter movement gained a new impetus when Rosenow, of Chicago, published a paper stating that systemic conditions of the heart, kidneys, muscles, joints, appendix and gall-bladder were due to infections, and that the organisms responsible for these were of the streptococcus variety. He stated further that these organisms gained entrance through the oral and nasal cavities and through the sinuses. Also that the same organism found in the systemic conditions were found in the dental abscesses and pyorrhea. The organism upon which most blame was laid was the streptococcus viridans.

We might say, then, that in the last ten years a new science or a new treatment has come to the fore in dentistry. Within that span of time many varied explanations, discussions and theories have been set forth, trying each in its own way to explain how and why focal infection has a direct bearing upon local and general disease.

What is focal infection? What does it mean? What is its significance in dentistry? These are some of the questions that arise in one's mind when the question first comes up.

Focal infection, according to the definition given by Dr. Bunting, is "a localized growth of organisms in tissues which either grow through the tissues or elaborate toxins which spread through the tissues."

This includes every part of the subject. Now let us proceed from there.

First of all, it is a localized growth of organisms in the tissues.

Second, these may grow through tissues to other tissues or elaborate toxins which may be distributed by the blood and lymph channels, as these are the only ways presumably that toxins have of being transferred to other tissues distant to the first infected focus.

In other words we have, *first*, foci of infection; *second*, foci for infection.

Is it true that an area infected by any group of organism can affect adjacent or distant tissues? Research proves that not only can the toxins be carried by the lymph and blood channels, but that the organism itself can also be transmitted that way, and that these may cause systemic diseases. The only qualifying statement as regards the latter is that the bacteria be in a sufficient quantity, and that if this is the case the invading bacteria will excite characteristic reactions in the infected tissues.

This latter statement must presuppose certain contingencies: first, that there be a general lowering of the bodily resistance; second, that there be susceptible tissues; third, that the organism be of a virulent type.

From what we know of immunity, we may deduct the following for the first contingency. When the bacteria or toxins enter the blood stream there are several types of reactions set up, all toward one purpose, that of destroying or warding off the invader. Among the most characteristic of the reactions we might mention leucocytosis, where the white blood cells go forth to battle with the invader, and the setting up of antibodies to neutralize the toxins that come in through the blood stream.

These two specific conditions would be below normal only when the whole bodily resistance was lowered. This is a vital factor and should at all times be considered.

Its dental significance? In this we will have to consider the entrance of the bacteria, their lodgment, their distribution, part of which we have already touched upon, and the quoting of specific cases to prove its relationship and significance to dentistry.

The following challenge to the dental profession was made by Dr. Charles Mayo in his famous statement at a dental meeting in Chicago, when he said, "The next great step in preventive medicine will come from the dental profession—will they take it?'' He little realized how great a stir he was going to raise. It only meant that the dental profession must take to its fundamentals now—to its histology, pathology and physiology to find the true solution of this focal infection query.

The main entrances of bacteria, according to Rosenow, are, if you will pardon the repetition, the nasal and oral cavities and their sinuses. In a consideration of this it seems as if we may further limit the entrance. In the first place, how are bacteria carried? Mainly by food and drink. The air has practically no pathogenic bacteria. Therefore we might readily say that the mouth is the main site. Once carried in by food and drink, they can be taken up by the following organs and tissues:

First, the tonsils.

Second, the mucous membranes of the mouth.

Third, the carious teeth.

The tonsils caused a great deal of trouble about ten years ago. This was the reason that then and practically up to the present time any one having any kind of a systemic disease was advised, in all seriousness, to have the tonsils removed. That, it was often explained to the poor suffering patient, was the cause of all his troubles. Many claims were set forth as to the benefits to be derived, but the cry of ''out with the tonsils'' only ceased with the other demand of the medical internists of out with the teeth.

The mucous membranes of the mouth are subdivided by Bunting into two distinct divisions:

1. The openings surrounding the teeth.

2. Those covering the cheeks, palate and alveolar process.

Normally the gum tissue is held tightly about the teeth, but a slight injury or irritation may loosen the tissues sufficiently to permit infection to travel to any of the tissues underneath.

In pyorrhea, the attachment of the mucous membrane to the tooth is cut away and we have formed a pyorrhetic pocket, a place where bacteria may grow unhindered and where they themselves, or their toxins, may spread to the rest of the body.

In a carious tooth the hard tissue is first broken down and then the bacteria destroy the pulp which becomes involved with it. This infection, after producing a putrefaction of the pulp, then passes out through the apical end of the root to infect and destroy the investing tissues with the formation of an abscess.

Having gained access to the mucous membranes about the teeth, and to those that are infected with organisms, how are the bacteria or the toxins carried from there? The teeth of both jaws and pericimentum, periosteum, alveolar process and gum tissue receive their blood supply from the external carotid artery through the branches of the internal maxillary artery.

The other mode of transmission is through the lymph. It was not many years ago that Dr. Noyes, of Northwestern University, showed that there was a lymph drainage from the alveolar process and the tooth pulps. He showed that there was a rich plexus of lymph vessels about the alveolus.

The different channels through which bacteria gain admission, if one can apply such a term to so minute an organism, are many and varied. One of the most important in relation to the systemic ills that follow dental diseases is probably that of pyorrhea alveolaris. Pyorrhea alveolaris is primarily a disease of the investing tissues of the teeth; that is, the gums, the gingiva, the alveolar process, the cementum and the pericementum.

This disease begins at the gingival border and progresses slowly towards the apical ends of the teeth, and if not arrested in time will result in a loosening and later to a loss of the teeth so affected.

What causes this condition? It may be the form or character of the organism, or the streptococcus or staphylococcus; and others believe it is the pneumococcus group, and still others claim that it is caused by the protozoan, the amoeba buccalis; and still others attribute the condition to all those already mentioned and add the spirochete and putrefactive organisms.

So you see how difficult the proposition is of ever clearing up this one focus of infection when no one has clearly demonstrated the cause definitely. The reason that so many people blame the streptococcus and staphylococcus is that nearly every disease infectious or otherwise is associated with their machinations or evil designs.

Second in importance is that of present uncertainty in root-canal therapy. One would be astonished at the many different opinions registered, not by those of inferior attainments or by those of the charlatan class, but by men upon whose integrity and scientific ability the dental profession has come to look with respect and admiration. In the past few years such men as Rhein, Best, Coolidge, Buckly and Calahan have stood out foremost in the rank of dental root-canal therapeutists. How many dental practitioners realize how many complicated operations there are involved in root-canal therapy? After deciding that a tooth should remain in the mouth, a formula something like this is generally put in practice:

1. The preparation of the patient involves, in the first place, a complete disinfection before opening the canal, which should be followed by the placing of the rubber dam.

2. Sterilization of the dressings and instruments.

3. Use of selected methods for opening canals.

4. Use of the different medicines and filling materials.

Periapical infection due to root-canal work gives the following opportunities for infection:

1. Contaminated canal and the canal walls and careless treatment.

2. Septic dentinal tubuli and difficulties of treatment.
3. Eroded apical cementum.
4. The granulation tissue investing the apex.
5. Diseased bone adjacent to a granuloma.

Why is it that, although we have so many good methods of opening canals and of filling them, and the means of correctly diagnosing the condition by the X-ray after filling, in the May issue of the *Dental Cosmos* of last year, Dr. Talbot, whom I am certain we all respect as a scientist, wrote, "My researches have shown that 95 per cent. of all roots are imperfectly filled, and it is a wonder to me that the other 5 per cent. do not show imperfections, and that after twelve years of research"?

The steps in the formation of an abscess and its sequelae are as follows:

1. There is an irritation. This may arise from a mechanical, a chemical or a bacteriological cause.
2. The mechanical inflammation is generally traumatic.
3. When bacteria enter the field with the inflammation, at first there is no exudate, but when the bacteria take possession then exudates in the form of toxins are thrown off.
4. Necrosis of the peridental membrane assures loss of the tooth.

Doesn't it seem as if one must get down to the bottom of this matter? To the fundemental principles that underlie all these procedures? And once you have a foci of infection started, what is to stop its ready ingress to the rest of the body? Doesn't it seem to you that one of the conclusions reached by Dr. Crane is the best answer so far given? He said that the establishment of scientifically correct culture methods and media is the crying need of the moment. Until this is accomplished the filling of the canal must be a probationary expedient.

And then again, what underlies this last principle? Just this, a thorough knowledge of histology and bacteriology and pathology.

Few of us realize the importance of Dr. Talbot's assertion. But when we follow it with the statement that oral foci of infection are located either at the root ends of the teeth, or at the gingival margins and the tissues adjacent to this area, you can then realize the significance of that remark.

To make a brief summary of focal infection, as it is related to root-canal work, you must consider two views:

The first one is that no devitalized tooth should be permitted to remain in the human mouth, because pulpless teeth must necessarily be infected ones, even though the vital pulp be removed under strict aseptic conditions as known or as practiced by our best operators, and the canals must be opened, sterilized and filled according to the best known technique.

There are sufficient examples mentioned to show the relationship that focal affection from dental abscess bears to the general health to make us reailze that there is danger of retaining an infected tooth.

On the other hand, there is the other view that the tooth from which a freshly devitalized pulp is removed can be treated and filled in such a manner that it is perfectly safe to keep in the mouth.

How many dentists, do you suppose, are filling root-canals that know their pathology? How many of us would wish to be treated by a physician who did not understand the pathological conditions of the body better than the average dentists do? I am certain few would risk it. Yet that is what, not the average, but more than the average dentist is doing to-day.

Dr. Bunting has given us this definition for a pathological condition: It is that condition which is abnormal —presupposing a knowledge of the normal. He further states that no organ or tissue of the body has a life history independent of the body as a whole. From that we may then deduce that pathologic reactions of the pulp fundamentally do not differ from the pathological reactions of the rest of the body. Instead of reading about the pathological reactions of the pulp, what do we find in the dental literature of to-day? Methods of filling, different opinions of filling materials, different methods of sterilization, when it is perfectly evident that this all really depends upon the operator. "The best method is the one which gives the best results in one's own hands." And we find that the most fundamental part, the most basic principle, is altogether left out, or practically so, that of pathology. This should take care of whether the canals should be filled or treated, and if the latter is undertaken accurate knowledge as to whether the pathological conditions of the underlying tissue have been restored to normal and the canals are really ready for filling. This, to my mind, is the most essential phase of this most complex situation.

Let us now leave the question of root-canal therapy and turn our attention to other dental operations in which the natural sequelae seem to be focal infection.

### BRIDGEWORK

Bridgework to-day has its troubles just like the drunkard who, coming home at 2 A. M., was met by his wife with the following: "Nice hour to arrive home and a nice state to arrive in. Explain, you brute!" "Old friend asked me to help him gather evidence of the war-time probition laws, and I jus' couldn't refuse," he answered.

There are as many adherents to the fixed kind of bridgework as there are to the removable kind—probably the latter is coming more to the fore. In an article in the *Pacific Dental Gazette* this is very ably discussed. I have taken the liberty of copying the following from it:

"Dr. Ottolengur, one of the foremost writers in the dental world to-day, said: 'Either the general practitioners of dentistry have already shown that they do not possess the requisite skill and innate ability to practice fixed bridgework, or else the disciples of this type of work will shortly be compelled to admit that fixed bridgework is wrong in principle. One horn of this dilemma the fixed bridgework man must ride—that is, that fixed bridgework as practiced to-day in this country is septic dentistry.' "

Dr. Burgess, a strong adherent and defender of the fixed type, has this to say: "I read in writings of the removable bridge advocate that the patient can take out his bridges and boil them. You say that you can get at them? Well, if you would use a small proportion of the time and the thought and everything else that you are putting into your removable bridges, you could make your fixed bridges so that you could get at them. It can be done."

Dr. Paul Stillman, of New York, has this to say: "In the twenty years of my practice I have observed that both the exquisitely executed and the crudely thrown together type of bridgework fail. By fail I mean that the tooth roots upon which they depended for their retention in the mouth become diseased; that the peridental membranes die from infection due to irritation caused by the bridge itself. I have seen removable bridges which were beautiful in perfection of their execution ruin a whole mouth, and within a comparatively short time have to be removed and full upper dentures made."

The reason that I have put down these three opinions was just to show that the whole question of bridgework did not depend after all upon beauty of construction, nor to perfection as to reproduction, but to the more fundamental principle—that of obtaining dental asepsis and the alleviating of focal infection. How many are there that have seen fixed or removable bridges that have not caused some irritation to the tissues? And what does irritation mean? Just this, that a focus of infection has been started.

For a summary of all dental operations and their relationship to oral focal infection, I can do no better than to turn to Dr. Hunter, who says that one of the worst cases of pernicious anemia was brought to him by a doctor, who when asked about the condition of the patient's mouth said that it had been carefully seen to and that it was in good form. The patient's mouth was one mass of gold caps, bridges, crowns, fillings, false teeth so well restored that an outsider could scarcely tell which were false and which real. The conditions of sepsis and necrosis revealed on the removal of this golden architecture was appalling. Gold fillings, crowns, bridges, fixed dentures, built in and around diseased teeth, form a veritable mausoleum of gold over a mass of sepsis to which there is no parallel in the whole realm of medicine and surgery. There is no rank of society free from the fatal effects on health of this surgical malpractice. He further goes on with this accusation: "The medical ill effects of this septic surgery are to be seen every day in those who are the victims of this guilded dentistry. In their dirty, gray, sallow, pale, waxlike complexions and in the chronic dyspepsias, intestinal disorders, ill health anemias and nervous complaints from which they suffer. In no class of patients and in no country are these more common than among Americans and in America, the original home of this class of work.

And Dr. Ottolengui has this to say: "Radiographers have pictured thousands of oral foci, 80 per cent. of which lurk under crowns and bridgework."

I believe that I have given sufficient authorities to show that focal infection is due in a very large degree to dental malpractice. What, then, has it to do with the rest of the body? It is true that the sequelae that I shall enumerate is directly traceable to this focal infection, or is it just another fad? Within the past twenty years we have had three so-called fads: one, that of removing all normal or diseased ovaries for nervous troubles; a second, the wholesale removal of all appendices; a third, the removal of all tonsils and adenoids, infected or otherwise.

From all the evidence that has been submitted thirty-one known diseases have been either directly or indirectly said to be caused by oral focal infection. Surely this, then, is not another surgical tomfoolery. The most important diseases attributed as sequelae to oral focal infection are chronic anemia, chronic arthritis, chronic nephritis, myocarditis, endocarditis, gastritis, colitis, inflammation of the joints, Bright's disease, weak heart, infection of the heart valves, nervous disturbances of all kinds ranging from mental depression up to actual lesions of the cord, and rheumatism.

Among the specific cases I have just taken a few that have been treated in our clinic. A senior dental student had rheumatism in his shoulder joints. Upon examination it was found that he had two small roots left from failure to extract all of a first molar. The roots were extracted and the rheumatism disappeared.

A very interesting case is reported by Dr. Rickert. One of his patients had a fistula on his hip four-fifths of an inch long. Dr. Darling had already been engaged to cut it away. Dr. Rickert suggested an X-ray of his mouth, and then they found that the man had an impacted third molar. Dr. Lyons performed the operation, the fistula closed. Dr. Rickert saw this man three years later and the fistula had up to that time not recurred.

Dr. Bunting reports several cases of men that could scarcely creep up the stairs even on crutches, yet after a general prophylaxis treatment he found the same patients a few days later walking up the college stairs. Many cases of anemia, gastritis and other diseases have been cured by the extraction of impacted third molars, or the treatment of abscesses, or by removing bridges, crowns, infected root-canals, etc.

Shall we turn to the other side of this question and say that focal infection and its relation to systemic infection has been greatly magnified, and without regard to all our theories? Can we say from the mass of evidence that is given that the beneficial results that follow the dental remedial measures must be explained upon other grounds? Can we say that the flora of pathological dental areas are low in virulence and can not therefore have much to do with general disease? Can we say that when bacteria or their toxins are discharged into the blood that a proportionate degree of immunity is obtained and no ill effects or results occur? Can we say that the treatment of a pyorrhea pocket, or the removing of root ends, or the removing of abscessed teeth, and other dental conditions that have primarily caused the infection, will not cure or aid in the cure of a secondary infected area? Yet there are many who still believe in these ideas. These men seem to have forgotten some general common truths, if I may be permitted the saying so.

Isn't it true that any infected area does increase the severity of another infected area in the same body? And isn't it also true that a mixing of bacterial flora causes the same thing? Therefore it may be stated that oral sepsis does increase the severity of any other infection, chronic or otherwise, that is located in any part of the body!

How are we to get rid of this condition? By what means can we eradicate, or at least tend toward doing so, in the field of dental operations? There are two means—one the curative, the other the preventative.

Dental preventative measures mean starting in with the teeth before the child is born—not at school, as most people have come to think. It is the duty of the attending family physician to instruct the mother or educate her, if you will, to the fact that the question of food makes for sound teeth in the child. There is no question of focal infection until the first teeth are erupted. The great measure that is to be taken care of in this respect is oral prophylaxsis.

The curative side has to deal with the medicinal treatment of the area infected. That means that, first, a correct diagnosis of the mouth must be had; second, a diagnosis as to focal infection and its relation to dental defects; third, the treatment of these defects must be complete.

The whole trouble lies in the second part—the curative one. When we were told about the up-to-date methods used in the examination of patients all the students in the class were surprised. Why should they be? The reason is that they have not thought nor has the average dentist been taught what a complete diagnosis means.

How many dentists think of carefully going over every tooth? How many of the dental practitioners ever think of radiographing the whole mouth? Too often the dentist just treats the tooth that needs to be treated immediately and leaves all others. How many men have urinalysis and blood examinations made of their patients who are suffering with focal infection? How many men take into consideration the general condition, which I have shown previously is so important a factor in the health of the patient? And this faulty neglect is not due always to the dentists themselves, but it is too often *the fault of their education.* It sometimes is the *college* or the teacher that is to blame. How many men when they graduate know how to interpret an X-ray film? For those who believe that they can do so, I'll just mention a few of the most important things that they should be able to tell: To determine whether the mouth is the focus of infection producing systemic disturbance. To determine whether the teeth are the cause of reflex irritation, such as neuralgia and headache. To determine whether there is apical or peridental infection. To determine pathological conditions of the alveolar process and the underlying tissues. To determine a perforation. To locate caries. To determine condition of the teeth before and after root filling, and when contemplating a proper restoration. You can see from this that a man must know his histology and pathology well to interpret an X-ray. From this it would seem that physiological chemistry, physiology and bacteriology, in addition to many other conditions unseen, must and should be taught, not only in the lecture room, but in the laboratory and in a clinic where these pathological conditions can be fully demonstrated.

If you will take a census of our so-called Class-A dental schools, in how many will you find complete courses in those fundamental studies that bear such a close relationship to the welfare of the body? What do most of us boast about? It can be summarized generally in the one word—technic. Therefore we can hardly expect the dentist to practice the curative method, for he is not fully prepared for such a contingency and must therefore rely on the preventative measures for keeping down focal infection.

### SUMMARY

1. That dentistry as it has been and is practiced to-day is not on a very sound basis when it comes to a practical knowledge of histology, bacteriology and pathology. Much of it is of the empirical type as was practiced not at all or during the Middle Ages.

2. That dentistry has been too much concerned with the restorative and architectural, and not deeply with the curative and scientific.

3. That too large a percentage of all root-canal work is a failure.

4. That 80 per cent. of all crown and bridgework leads to focal infection sooner or later.

5. That dental focal infection may be the direct cause of certain systemic diseases.

6. That dental focal infections are of the worst type and of the greatest menace to the individual because:

(*a*) Asepsis is almost impossible.

(*b*) Focal infection does not give any local pain or discomfort.

(*c*) That foci exist about roots of teeth supposed to be well filled.

(*d*) Systemic effects are produced so gradually that an ordinary individual will not be conscious of the deviation of the normal.

(*e*) Often infection exists about the roots of septic teeth without giving evidence of their presence radiographically.

(*f*) That any focal infection due to oral conditions in any person having infections of another kind does increase this later, whether due to the same or different organisms.

7. That the curricula of the dental colleges should be enlarged so as to include physiology, bacteriology and pathology, in both lectures, laboratories and clinical work.

8. That students must be taught to be interested as much in the curative and preventative measures as in the restorative measures of dental art.

9. That fundamental training should be equivalent to that of the students of medicine before any aid can be had in the control of focal infection.

